# Diarrhœa in Children

**Published:** 1893-09

**Authors:** 


DIARRHŒA IN CHILDREN—*Drugs in.*—At the New York Academy of Medicine on May 12, 1892, Dr. J. Milton Mabbott said: Until recently there seems to have been general consent to the administration of alkalies. But now that we endeavor to promote asepsis and control fermentation by evacuant, dietary, and hygienic measures, they are certainly less important than formerly. They are usually given with or soon after feeding. When using pepsin, alkalies should be given midway between feedings. The indications for acids are doubtful. Lactic acid, as proposed by Hayem, is advocated in (1) acute infectious diarrhœa where the stools are numerous, watery, and often foul but yellow in color; and (2) in green bacillary diarrhœa, for which it is recommended as a specific. Numerous observers have found the reaction of the alimentary canal in healthy infants acid throughout; and Pfeiffer has shown that green stools are associated with alkalinity. Hence, the use of acids would seem to have a rational basis. The dilute mineral acids are commended by many, the dose being one to five drops administered twenty minutes after feeding. The vegetable astringents have during the last few years been almost discarded. The same is true also of mineral astringents, with a single exception. That exception is bismuth, the subnitrate being the preparation universally esteemed. It is prescribed in much larger doses than formerly, twenty grains every two hours sometimes being given to an infant. Opiates are less used than formerly. They undoubtedly check peristalsis. As peristalsis is increased in diarrhœa, this action is desirable after the bowels have been emptied of their objectionable contents, but highly dangerous before. The other indications for opium are relief of restlessness, pain and tenesmus, and the control of frequent watery passages. Ashby and Wright recommend it in the latter stages, if the passages continue small and numerous. Holt and Crandall always prescribed the opiate separately, so that it may be conveniently increased, diminished or withheld at will; for increasing fever or taxic symptoms call for its discontinuance. It should not be given when the passages are infrequent and of bad odor. A decrease in the number of stools while they become more offensive, contraindicates its use and demands evacuants.—*Boston Medical and Surgical Journal.*

